# Differential activation of serotonergic neurons during short- and long-term gregarization of desert locusts

**DOI:** 10.1098/rspb.2014.2062

**Published:** 2015-02-07

**Authors:** Stephen M. Rogers, Swidbert R. Ott

**Affiliations:** 1 School of Biological Sciences, University of Sydney, A08 Heydon-Laurence Building, New South Wales 2006, Australia; 2 Department of Biology, University of Leicester, Adrian Building, University Road, Leicester LE1 7RH, UK

**Keywords:** 5-HT, phase change, neuromodulation, behavioural plasticity, phenotypic plasticity

## Abstract

Serotonin is a neurochemical with evolutionarily conserved roles in orchestrating nervous system function and behavioural plasticity. A dramatic example is the rapid transformation of desert locusts from cryptic asocial animals into gregarious crop pests that occurs when drought forces them to accumulate on dwindling resources, triggering a profound alteration of behaviour within just a few hours. The onset of crowding induces a surge in serotonin within their thoracic ganglia that is sufficient and necessary to induce the switch from solitarious to gregarious behaviour. To identify the neurons responsible, we have analysed how acute exposure to three gregarizing stimuli—crowding, touching the hind legs or seeing and smelling other locusts—and prolonged group living affect the expression of serotonin in individual neurons in the thoracic ganglia. Quantitative analysis of cell body immunofluorescence revealed three classes of neurons with distinct expressional responses. All ganglia contained neurons that responded to multiple gregarizing stimuli with increased expression. A second class showed increased expression only in response to intense visual and olfactory stimuli from conspecifics. Prolonged group living affected a third and entirely different set of neurons, revealing a two-tiered role of the serotonergic system as both initiator and substrate of socially induced plasticity. This demonstrates the critical importance of ontogenetic time for understanding the function of serotonin in the reorganization of behaviour.

## 1. Introduction

Serotonin (5-hydroxytryptamine) is a monoamine neurotransmitter, neuromodulator and neurohormone that has a prominent and evolutionarily conserved role in the control and regulation of a wide range of behaviours [1,2]. Serotonin can influence the state of much of the central nervous system (CNS), affecting suites of linked behaviours such as the regulation of feeding and diet [3–5], arousal [6], sleep and circadian activity [7–9], all of which can substantially alter the interactions between an animal and its environment. In particular, serotonin is strongly associated with how animals engage and cope with their social environment [10–13]. For example, it modulates intra-specific aggression and other behaviours associated with social status and the establishment of dominance hierarchies in vertebrates [14–17], crustaceans [18–21] and insects [22–24].

**Figure 1.**
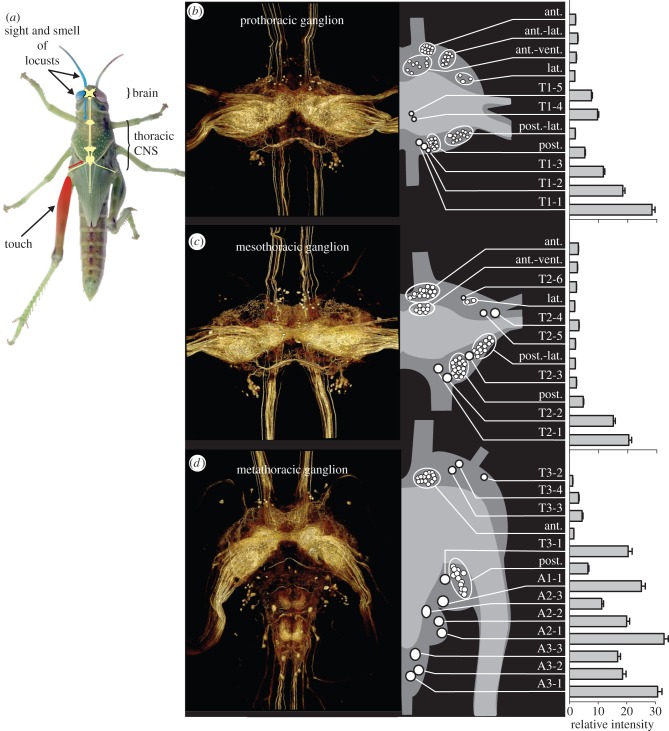
Serotonergic neurons in the thoracic ganglia. (*a*) Last larval instar solitarious locust showing the CNS and the two separate sensory pathways by which behavioural gregarization can be induced: a thoracic pathway (red) activated by touch stimuli directed to a hind femur, and a cephalic pathway (blue) activated by the combined sight and smell of other locusts. (*b–d*) Expression of serotonin in the (*b*) prothoracic, (*c*) mesothoracic and (*d*) metathoracic ganglion. For each ganglion, the image on the left shows a three-dimensional rendering of the total serotonin immunofluorescence in a whole-mount preparation; the schematic drawing gives the locations and sizes (at 2× scale) of the serotonergic somata; and the bar plots show the mean intensity and s.e.m. for each soma or soma cluster as a multiple of the mean neuropile intensity (*n* = 55).

Serotonin also has a pivotal role in the initial behavioural transformation of the desert locust (*Schistocerca gregaria*) from a solitarious phase of living alone to a swarming gregarious phase of banding together that is characterized by rich and varied social interactions [25,26]. This transformation provides a powerful model with which to analyse both the short- and long-term effects of serotonin, as the two phases differ profoundly in behaviour, physiology and neurochemistry [27–31]. Solitarious locusts actively avoid one another, thereby dispersing themselves thinly across the environment. They have camouflage coloration, walk with a low creeping gait [29], have a narrow dietary range [32] and are crepuscular in habit. Increasing population density triggers the transformation to the gregarious phase, which is directly induced by stimuli from other locusts in close proximity. There are two separate sensory pathways by which this may happen: a mechanosensory pathway activated by touch stimuli to the hind femora, and a combined visual and olfactory pathway driven by the sight and smell of other locusts [33–35] (figure 1*a*). After as little as 4 h of exposure to these stimuli their behaviour has become quantitatively indistinguishable from locusts that have been in the gregarious phase for many generations: these previously solitarious locusts become much more active, walk rapidly with the body held high off the ground, lose their mutual repulsion and instead become attracted to each other, forming coherent groups [26,29,36]. This leads to the continual exposure to stimuli from other locusts, which drives the process of transformation onwards through positive feedback. In time, gregarious locusts acquire bright aposematic warning colours [37,38], change their dietary regime [32,39] and become more strongly diurnal, and interactions with other locusts become more complex and agonistic [40].

The onset of exposure to gregarizing stimuli leads to a dramatic increase in the amount of serotonin in the thoracic ganglia but not in the brain, as measured by HPLC [30] (figure 1*a*), which is sufficient and necessary to induce the initial transformation from solitarious to gregarious behaviour [25,26]. This increase in serotonin is transient, however, decreasing again within 24 h, and in the longer term, adult gregarious locusts have approximately half the amount of long-term solitarious locusts in their thoracic CNS [30]. Two lines of evidence indicate that serotonin acts centrally within the thoracic ganglia, not hormonally. First, the thoracic ganglia lack efferent serotonergic neurons [41]; and second, applying serotonin locally to the thoracic ganglia induces gregarious behaviour and focally injecting receptor antagonists into the thoracic ganglia blocks gregarization. Despite the critical function of serotonin in behavioural gregarization, the locust CNS contains few serotonergic neurons [41], and these few neurons must first orchestrate the rapid transition to gregarious behaviour and then function in the altered regulatory regime in relation to diet, activity and circadian patterns that arises as a consequence of this transformation. This raises two questions. First, do the two independent gregarizing pathways induce all the serotonergic neurons in the thoracic CNS to increase synthesis or is the change confined to particular subsets? Second, is the longer-term decrease in serotonin brought about by changes in expression in these same neurons?

To answer these questions, we analysed how acute crowding, acute activation of either of the two gregarizing pathways and prolonged group living each affect the expression of serotonin in individual serotonergic neurons in the thoracic ganglia. The rationale for this experimental design is that neurons that respond equally to crowding and to stimulation of either of the two pathways in isolation are likely to be causally involved in behavioural gregarization, whereas neurons that respond to only one pathway may have roles in other contexts. This analysis revealed that, in the early phase of behavioural transformation, stimuli from conspecifics recruit a specific subset of neurons to upregulate serotonin, whereas prolonged group living causes a downregulation of serotonin in a separate population of serotonergic neurons.

## 2. Material and methods

### (a) Locusts

Locusts were obtained from colonies maintained at the Department of Zoology, University of Oxford, UK. Long-term gregarious locusts were taken directly from stock that had been reared in large plastic bins (56 × 76 × 60 cm) at high population density (450–1000 insects per bin) for many generations. Long-term solitarious locusts were obtained from this long-term gregarious stock by rearing them in isolation for three generations using the husbandry regime detailed in [42]. Both colonies were maintained under a 12 L : 12 D photo regime at 30 ± 2°C and on a diet of fresh wheat seedlings and wheat germ. Final larval instar locusts were used in all experiments.

### (b) Treatments and behavioural assay

A minimum of 25 locusts each were subjected to one of five experimental treatments (see the supplemental methods in the electronic supplementary material for details): (i) long-term solitarious, (ii) long-term gregarious; the remaining three treatments entailed exposure for 1 h of long-term solitarious locusts to one of three different gregarizing regimes: (iii) crowding with 20 gregarious locusts in a small cage, (iv) touching repeatedly on a hind femur [34], and (v) intense visual and olfactory stimulation (sight + smell) from 500 to 1000 gregarious locusts but no physical contact [33] (see the electronic supplementary material for further details). After treatment, the behavioural phase state of each individual locust was assessed in an arena-based assay [42]. Immediately after behavioural observation, the thoracic ganglia were rapidly dissected under ice-cold physiological saline and fixed using a method modified from Ott [43], as detailed in the supplemental methods in the electronic supplementary material. The behavioural data were analysed using the logistic regression model of Anstey *et al*. [25] to obtain a single probabilistic metric of gregarious behaviour, *P*_greg_, which runs from 0 for fully solitarious to 1 for fully gregarious. Only long-term gregarious locusts with a measured *P*_greg_ > 0.95, long-term solitarious locusts with a *P*_greg_ < 0.05 and acutely treated locusts that had gregarized to *P*_greg_ > 0.9 were selected for further processing. This yielded a sample size of 12 for each of the treatment groups except for the sight + smell-treated group, in which only seven animals had become sufficiently gregarious.

### (c) Serotonin immunofluorescence staining and imaging

The fixed ganglia were whole-mount stained with a polyclonal rabbit anti-serotonin antiserum (Sigma; 1 : 4000) and Cy3-conjugated goat anti-rabbit IgG antibodies (Jackson ImmunoResearch; 1 : 200) following the protocol of Ott [43] with some modifications. Stacks of confocal images were captured with a 10× objective (NA 0.4, pin hole size 1.0 AU). See the supplemental methods in the electronic supplementary material for details and validation of the specificity of the immunostaining. We calculated the theoretical optical slice thickness (full width at half maximum, FWHM) following [44]:4.1
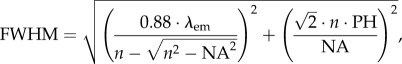
which gives FWHM = 14.25 µm with emission wavelength *λ*_em_ = 570 nm, refractive index *n* = 1.52, numerical aperture NA = 0.4 and a back-projected object-side pinhole diameter PH = 2 µm. Assuming that the *z*-resolution in whole-mounted tissue is 1.5× lower (FWHM approx. 21 µm), we acquired confocal sections at a mechanical *z*-step of 7 µm to give an optical inter-slice distance of 11 µm [45] so that adjacent confocal sections overlap by an estimated 50% FWHM.

### (d) Image analysis

Image stacks were analysed in ImageJ software (NIH, USA; http://rsb.info.nih.gov/ij/). Large somata could be easily identified in all individuals as bilateral pairs by their size and position in the ganglion. For each soma, we measured the mean pixel brightness along two orthogonal line transects that spanned the whole cell body in the single optical section that was closest to the mid-plane of the cell body. The two measurements were averaged to give a mean intensity per cell, and the final value used for analysis was the mean of the left and right soma. Somata that were part of a cluster could not be individually identified. Each of these neurons was measured using a single line transect to obtain an average brightness value across the soma. The cluster as a whole was characterized by the sum of these values. To control for the stochastic variation in antibody penetration between preparations, we used the total integrated brightness of the neuropile as a covariate in our analyses of treatment effects. The total integrated neuropile brightness was measured after non-rigid registration of each individual ganglion to a reference ganglion [46] (CMTK; http://www.nitrc.org/projects/cmtk/) and applying a common mask to isolate the neuropile. There were no significant differences in total neuropile brightness in any of the three thoracic ganglia between the five experimental treatments (MANOVA; Wilks's *λ* = 0.699, *F*_12,127.3_ = 1.741, *p* = 0.065; for prothoracic ganglion *F*_4,50_ = 0.478, *p* = 0.775; for mesothoracic ganglion *F*_4,50_ = 1.441, *p* = 0.243; for metathoracic ganglion *F*_4,50_ = 0.478, *p* = 0.751).

### (e) Statistical analysis

Multivariate analysis of variance (MANOVA) and covariance (MANCOVA) were carried out in PASW Statistics v. 18 (Polar Engineering and Consulting, USA). The effects of treatment on individual neurons were analysed by comparing the intensities of staining in the long-term gregarious, crowded, touch and sight + smell stimulated locusts in independent contrasts against the long-term solitarious locusts (electronic supplementary material, tables S1–S3). Values are given as means ± s.d.

## 3. Results

Serotonin immunofluorescence staining revealed a small population of neuronal somata in each of the three thoracic ganglia and densely interwoven neuronal processes that ramified throughout the neuropiles (figure 1). These processes arose from a mixture of interneurons with somata in the thoracic ganglia, intersegmental interneurons with somata in other parts of the CNS, and some incoming sensory afferents. The latter made the ventral association centres (VACs) particularly well delineated. The serotonergic neurons occurred as mirror-symmetrical left–right pairs in each ganglion. In the following, the counts refer to just one half of a ganglion. Many interneurons could be recognized as individual identified cells across all preparations from the characteristic locations, diameters and relative brightness of their immunopositive somata (figure 1*b–d*). These were given individual names. Other cells occurred in clusters of 3–17 similar somata that could not be reliably separated by these criteria; each of these clusters was treated as an aggregate entity with the sum staining intensity of individual somata in the entire group used in the analyses.

The prothoracic ganglion (T1) contained five individually identifiable somata, five clusters each containing 9–13 somata, and one small cluster of three somata (table 1 and figure 1*c*). The mesothoracic ganglion (T2) contained six individually identifiable somata, four clusters of 13–17 somata and one pair of neurons that could not be individually distinguished (table 1 and figure 1*d*). The metathoracic ganglion contained 11 identifiable somata across its four constituent neuromeres, with four in the metathoracic neuromere (T3), one in the first abdominal neuromere (A1) and three each in A2 and A3 (table 1 and figure 1*e*). There were just two soma clusters in this ganglion, both in T3: one next to the anterior connectives and the second adjacent to the boundary where T3 joins A1. The most intensely stained somata were found in the caudal cortex of each neuromere. These somata were 15–30× brighter than the mean intensity of staining in the neuropile. The remaining individually identifiable somata were fainter, 1–5× brighter than the mean neuropile intensity, with the somata in the clusters the most weakly stained ones.

**Table 1.** Results of multivariate analyses of variance (MANOVA) of cell body immunofluorescence intensity for each soma or cluster of somata in each of the three thoracic ganglia, using total integrated brightness of the neuropile as covariate and treatment group as main effect. Please refer to the material and methods section for how total neuropile brightness was calculated. Significant effects of treatment are shown in bold.

metathoracic ganglion						mesothoracic ganglion						prothoracic ganglion					
MANOVA — all neurons						MANOVA — all neurons						MANOVA — all neurons					
*neuropile*			*treatment*			*neuropile*			*treatment*			*neuropile*			*treatment*		
*λ*	*F*_13, 37_	*p*-value	*λ*	*F*_44, 151_	*p*-value	*λ*	*F*_11,39_	*p*-value	*λ*	*F*_44,151_	*p*-value	*λ*	*F*_11,39_	*p*-value	*λ*	*F*_44,151.2_	*p*-value
0.194	11.81	<0.001	0.079	2.598	**<0.001**	0.154	19.52	<0.001	0.151	2.191	**<0.001**	0.204	13.86	<0.001	0.191	1.859	**0.003**

ANOVAs — individual neurons						ANOVAs — individual neurons						ANOVAs — individual neurons					
		*neuropile*		*treatment*				*neuropile*		*treatment*				*neuropile*		*treatment*	
*neuron*		*F*_1,49_^a^		*F*_4,49_	*p*-value	*neuron*		*F*_1,49_^a^		*F*_4,49_	*p*-value	*neuron*		*F*_1,49_^a^		*F*_4,49_	*p*-value
A3 cell 1		61.23		4.658	**0**.**003**	T2 cell 1		66.23		4.086	**0**.**006**	T1 cell 1		35.21		2.230	0.079
A3 cell 2		58.07		5.422	**0**.**001**	T2 cell 2		75.09		3.012	**0**.**027**	T1 cell 2		25.40		0.419	0.794
A3 cell 3		77.07		6.678	**<0**.**001**	T2 posterior group		63.53		3.735	**0**.**010**	T1 cell 3		23.51		0.341	0.849
A2 cell 1		65.72		3.831	**0**.**009**	T2 cell 3		53.70		3.618	**0**.**012**	T1 posterior group		23.42		0.427	0.789
A2 cell 2		66.36		7.865	**<0**.**001**	T2 posterolat. group		27.60		1.492	0.219	T1 posterolat. group		124.8		2.206	0.082
A2 cell 3		103.3		5.726	**0**.**001**	T2 cell 5		61.89		1.648	0.177	T1 cell 4		56.62		2.138	0.090
A1 cell 1		101.0		8.523	**<0**.**001**	T2 cell 4		58.36		9.085	**<0**.**001**	T1 cell 5		33.82		0.982	0.426
T3 lateral group		82.05		6.897	**<0**.**001**	T2 lateral pair		112.5		5.733	**0**.**001**	T1 lateral group		87.77		3.346	**0**.**017**
T3 cell 1		88.03		3.059	**0**.**025**	T2 cell 6		82.70		2.831	**0**.**034**	T1 anterolat. group		16.96		4.141	**0**.**006**
T3 cell 2		41.24		2.964	**0**.**029**	T2 anterovent. group		26.43		5.423	**0**.**001**	T1 anterovent. group		31.78		2.365	0.066
T3 cell 3		92.06		3.516	**0**.**013**	T2 anterior group		26.70		6.651	**<0**.**001**	T1 anterior group		42.82		4.845	**0**.**002**
T3 cell 4		76.70		0.840	0.507												
T3 anterior group		60.26		2.966	**0**.**028**												

^a^all *p* < 0.001.

**Figure 2.**
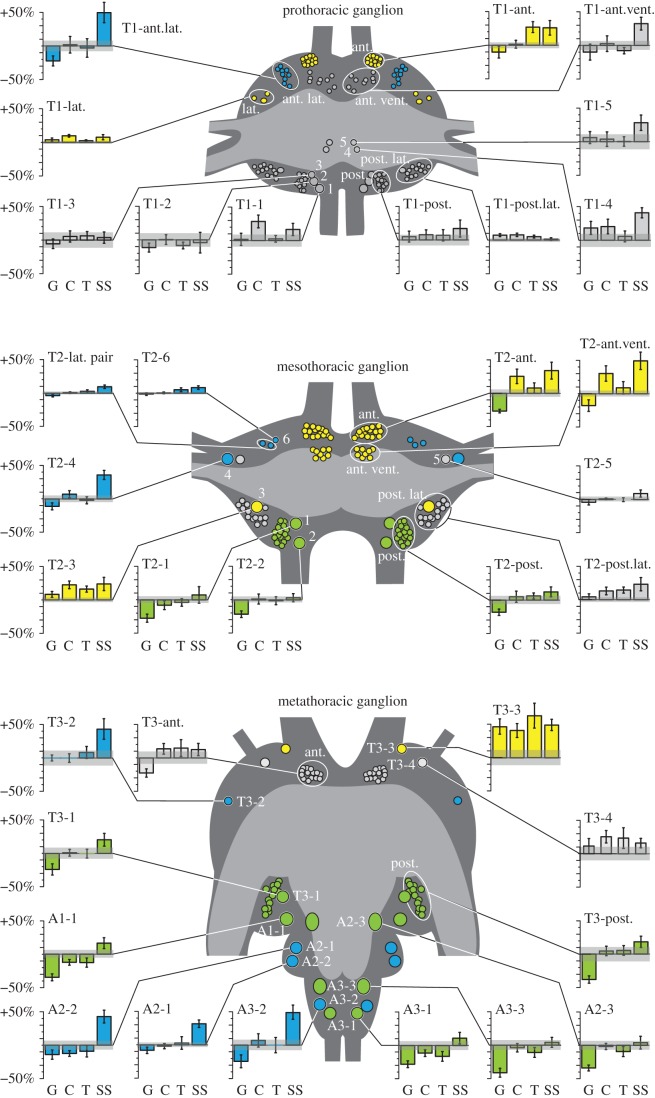
The responses of serotonergic neurons in the thoracic ganglia to different gregarizing treatments: long-term gregarious (G; *n* = 12); crowded with 20 other locusts for 1 h (C; *n* = 12); tickled on a hind femur for 1 h (T; *n* = 12); and exposed to the sight and smell of a gregarious colony cage for 1 h (SS; *n* = 7). The bar plots show the percentage change in serotonin immunofluorescence relative to mean long-term solitarious values and the grey bar gives the s.e.m of that value (*n* = 12). Neuronal somata are shown at 2× natural size and are colour-coded according to effects of treatment: green, significantly different in long-term gregarious locusts; blue, significantly brighter in locusts exposed to the intense sight and smell of other locusts; yellow, affected by two or more of the gregarizing treatments. Somata in grey were no different under any condition.

To test whether any of these identified single somata or clusters differed in serotonin expression across the five experimental treatments, we devised a statistical analysis that corrects for the non-biological variation in overall brightness between preparations: we measured the total integrated brightness of the neuropile in each preparation and used this as a covariate in a MANCOVA. This revealed that 23 out of 35 identified individual somata or clusters across the three ganglia differed significantly in immunofluorescence brightness across two or more of the five treatments (figure 2 and table 1). The somata affected by treatment fell into three distinct groups: (i) increased expression in response to more than one of the gregarizing stimuli, (ii) increased expression after exposure to the sight + smell stimulus only and (iii) decreased expression in long-term gregarious locusts but unaffected by the gregarizing stimuli in the short term.

### (a) Neurons affected by multiple gregarizing stimuli

**Figure 3.**
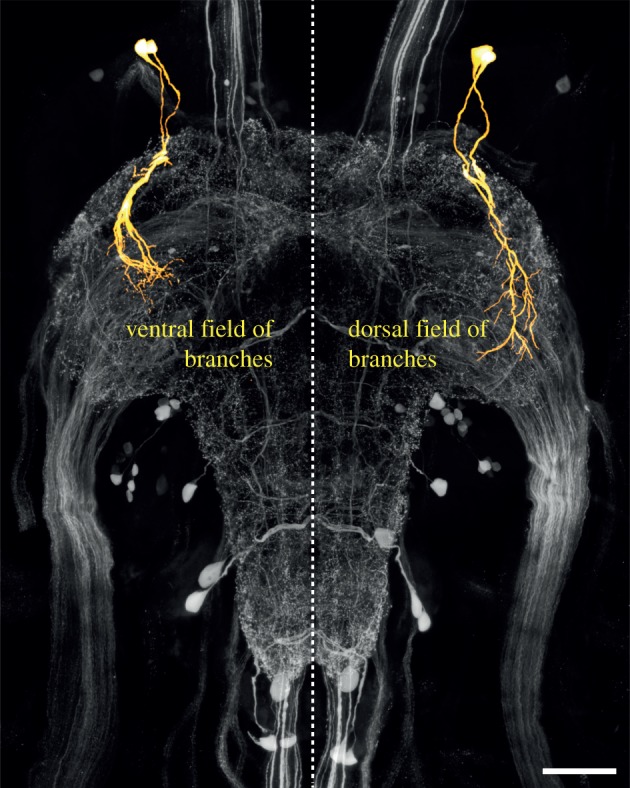
Partial reconstruction of a pair of neurons (T3-3 and T3-4) in the metathoracic ganglion that show increased serotonin expression following exposure to all gregarizing stimuli. The neurons have a ventral field of branches in the VAC and a dorsal field of branches projecting along the dorso-lateral edge of the neuropile. The reconstructions (yellow) are superimposed on a projection view of the serotonin immunofluorescence (grey) in the entire ganglion. In the reconstructions, the dorsal field of arborizations has been omitted in the left half of the ganglion (to show only the somata, primary neurites and ventral field), and the ventral field of arborizations has been omitted in the right half of the ganglion (to show only the somata, primary neurites and dorsal field).

All three ganglia contained serotonergic neurons that were more intensely stained in locusts physically crowded with 20 other locusts or subjected to the mechanosensory or sight + smell gregarizing stimuli for just 1 h (yellow somata in figure 2; independent contrasts of the effect of treatment are given in the electronic supplementary material, tables S1–S3). In the metathoracic ganglion, there was a single soma (T3-3) adjacent to the anterior connectives that was brighter in all gregarious locusts, irrespective of whether newly gregarized or long-term gregarious (range: +39 ± 10% to +63 ± 19% brighter than in long-term solitarious). This neuron is the brighter of a pair whose primary neurites enter the neuropile near the root of nerve 1. The dimmer neuron of this pair (T3-4), while not showing a significant difference, showed a similar differential pattern of staining, being between 11 and 26% brighter in all behaviourally gregarious locusts. These neurons had two major fields of branching (figure 3): a dorsal field near the lateral edge of the neuropile and a ventral field of branches in the VAC, the projection region of incoming mechanosensory afferents from the hind leg [47]. Electronic supplementary material, figure S3 shows the staining intensity in these neurons in six uncrowded locusts, six locusts that had been crowded for 1 h and six that had their hind leg stroked for 1 h.

Neurons showing a response to multiple gregarizing treatments were more numerous in T2. Adjacent to the anterior connectives there was a tight cluster of 15 ± 0.3 somata (T2-anterior group), in a similar location to the metathoracic pair of T3-3 and T3-4. This cluster stood out as the only instance where neurons were significantly brighter in locusts subjected to the short-term crowding (+25 ± 10.5%) and the sight + smell (+34 ± 12.3%) treatments, but fainter in long-term gregarious locusts compared with untreated solitarious locusts (–20 ± 6.7%). T1 contained a similar tight grouping of 13 ± 0.4 somata in an equivalent location (T1-anterior group) that was brighter in locusts given either the touch (+27 ± 8%) or the sight + smell (+26 ± 10.9%) treatments but was not different in long-term gregarious locusts. Another grouping of somata in the anterior cortex, but in a more ventral and slightly more posterior location, was found in all three ganglia (T3-anterior group, T2- and T1-anteroventral groups; consisting of 14 ± 0.3, 9 ± 0.4 and 8 ± 0.3 somata, respectively). In T2, these neurons were more brightly stained in locusts subjected to the crowding (+27 ± 10%) and sight + smell (+46 ± 12%) stimuli, but there was no significant effect of any treatment on the corresponding neurons in T1 or T3. A single neuron (T2–3) amid a cluster of smaller, more weakly stained neurons (T2-posterolateral group) in the posterolateral cortex of T2 was brighter in locusts given any of the short-term gregarizing treatments (range +16 ± 4.5 to +23 ± 9.9% brighter), but in long-term gregarious locusts it was no brighter than in solitarious locusts. This neuron had no apparent serial homologue in the other ganglia.

### (b) Neurons affected by the sight and smell of other locusts

These neurons were found in all three ganglia and showed a pronounced increase in staining intensity in solitarious locusts that had been subjected to the intense sight and smell of gregarious locusts only but were unaffected by any of the other treatments (blue somata in figure 2; independent contrasts of the effect of treatment are given in the electronic supplementary material, tables S1–S3). In the metathoracic ganglion, particularly intensely stained neurons occurred in A3 (A3-2, +49 ± 10.1%) and A2 (A2-1, +43 ± 10%; A2-2, +24 ± 5.4%; figures 1*d* and 2*c*). Other somata showing the same pattern were: a much fainter anterolateral soma in T3 (T3-2, +42 ± 15%; figure 1*d*) and a soma in a similar location in T2 (T2-4, +35 ± 6.6%; figure 1*c*); three anterolateral somata in T2, two of which could not be distinguished by intensity or diameter (T2-6, +18 ± 8.5%; T2-lateral pair, +10 ± 2.5% brighter); and a cluster of 9 ± 0.35 somata in T1 that formed a distinct band in the anterolateral cortex (T1-anterolateral group) with no clear equivalents in the other ganglia (+50 ± 15.0% brighter).

### (c) Differences between long-term solitarious and long-term gregarious locusts

The third group were those neurons that showed less serotonin immunofluorescence in long-term gregarious locusts than in solitarious locusts, yet were unaffected by any of the gregarizing treatments in the short term (independent contrasts of the effect of treatment are given in the electronic supplementary material, tables S1–S3). These neurons were confined to the meso- and metathoracic ganglia (green somata in figure 2), and included the large neurons in the posterior cortex (T2-1, T2-2; T3-1; figures 1 and 2) and the cluster of smaller neurons associated with them (T2-posterior group and T3-posterior group; range between −38 ± 5.3% and −18 ± 4.7% from mean long-term solitarious value). The staining intensity of serotonergic neurons in equivalent positions in T1 (T1-1, T1-2, T1-3 and T1-posterior group) was not affected by any of the treatments. In the abdominal neuromeres of the metathoracic ganglion, the largest neurons (A1-1, A2-3 and A3-3) and one of the paired neurons in A3 (A3-2) were less bright in long-term gregarious locusts (range between −41 ± 6.6% and −28 ± 5.1% of solitarious values).

## 4. Discussion

Locusts have a small population of serotonergic neurons in their thoracic CNS, and our data indicate that of these, only a subset mediate the surge in serotonin production that initiates behavioural gregarization. The most intensely stained of these neurons were all located in the same relative location just ventral and lateral to the anterior connectives of each of the three thoracic ganglia. In the pro- and mesothoracic ganglia, these occur as a population of 13–15 neurons on each side, but in the metathoracic ganglion only a pair of neurons in a similar location was present. A completely distinct set of neurons in the thoracic population seems to account for the difference in serotonin synthesis in the fully established phases, with one exception (T2-anterior group). Almost all neurons that differed between the fully established phases had less intense anti-serotonin staining in long-term gregarious locusts, agreeing with our previous finding that serotonin titres, as measured by HPLC, were lower in the CNS of locusts showing the full gregarious phenotype than in solitarious locusts [30]. Changes in the intensity of staining in the somata are probably a conservative measure of changes in serotonin synthesis, since most synthesis and release will occur towards the terminals of the neurons in the neuropile. If the changes we see were to result solely from a redistribution of serotonin within neurons, however, then no overall change in serotonin would have been detected by the previous HPLC studies [25,30]. The changes in soma expression are therefore likely to be a passive reflection of changes in overall serotonin synthesis, although redistribution of serotonin within neurons might additionally occur as part of the changes that occur during phase transition. The extensive and highly intertwined arborizations in the neuropile, however, currently make it impossible to analyse serotonin expression in distal processes and relate it to individual neurons. Furthermore, the many serotonergic afferent fibres that enter the thoracic ganglia through the lateral nerves [41] may also respond directly to tactile stimuli that induce gregarious behaviour. Nevertheless, the present findings provide targets for intracellular recording and dye injection to further analyse their function and structure.

The neurons that only responded to the sight + smell stimuli from other locusts are perplexing, since the crowded locusts should have received qualitatively comparable stimuli. The level of exposure may account for the difference: the crowded locusts were exposed to only 20 other locusts in a well-ventilated room; those given the sight + smell treatment were exposed to hundreds of other locusts in the gregarious colony room, which is permeated with a strong locust odour. Increased serotonin synthesis in these neurons may therefore not be directly related to gregarization but linked, for example, to changes in activity or ventilation rate brought about by the stressful experience of suddenly being exposed to so many locusts. Serotonin can elicit changes in neuronal function that protect against heat and other environmentally induced shocks, and it specifically protects the ventilatory system of locusts in this manner [48]. The largest and most intensely stained serotonergic neurons affected by the sight + smell treatment occurred in the metathoracic ganglion, where much of the central pattern generator for ventilation is located [49,50]. At present, it is unknown whether these neurons are activated only by the combination of visual and olfactory stimuli from other locusts, or whether intense stimuli of either modality suffice.

The neurons that differed between long-term solitarious and gregarious locusts were confined to the meso- and metathoracic ganglia. These ganglia control major motor systems controlling flying, jumping and walking [50], and long-term differences in serotonin expression are therefore likely to reflect phase-specific sensorimotor requirements (e.g. [51]). Little is known about the role of serotonin within the thoracic circuitry, except that exogenously applied serotonin modulates a synapse between two motor neurons in gregarious locusts [52]. Even though this study was conducted on final instar nymphs lacking full wings it is likely that the serotonergic system anticipates the adult condition. The wings lie over and conceal most of the body when at rest and therefore form a primary tactile interface with other locusts [53,54]. Locusts will respond to tactile stimulation of the wings with directed scratching movements [53–56], which are important in repelling the agonistic attentions of other locusts [40]. In cockroaches, serotonin applied to the thoracic ganglia decreased the responses of leg motor neurons to tactile stimulation of the cerci [57]; it is possible therefore that the decreased expression of serotonin in long-term gregarious locusts makes them more responsive to tactile stimuli and thus repel other locusts.

Elevated serotonin is not a marker of gregariousness *per se* and gregarious behaviour is not maintained by continual high exposure to serotonin [30]. With respect to behaviours such as posture, activity, locomotion and attraction towards conspecifics, newly gregarized locusts closely resemble those that have been gregarious for generations [26,29], yet as we show here, their cellular profiles of serotonin expression are very different. With only one exception (T3-3), serotonergic neurons were different either between the fully established phases or transiently during gregarization, but not both. This implies that a set of phenotypically similar behaviours can be under very different neuromodulatory control depending on the *stage* of the transformation process. To maintain the new behavioural phenotype in the longer term, changes in gene expression and structural remodelling of the neuronal circuits that organize the phenotype need to substitute for the labile modifications brought about by neuromodulators. These more profound changes may in turn free neuromodulatory neurons to take on new regulatory roles in the fully established phenotype. This may in fact be a necessary requirement, since vertebrates, arthropods and other invertebrates all typically have very few serotonergic interneurons with wide-ranging ramifications throughout much of the CNS [41,58–63] that are required to undertake a wide range of regulatory roles. In vertebrates, different populations of dorsal raphe serotonergic neurons have very different transcriptomes [64], but in general little is known about the functional sub-division of serotonergic neurons. Our data suggest that there may be a hierarchy of plasticity: a global reconfiguration of the CNS for a new behavioural state (here, the onset of behavioural gregarization) demands new regulatory regimes for the behaviours that are altered as a consequence of this change (such as diet or circadian activity), all of which may themselves have a serotonergic component. Such a tiered change in a neuromodulatory regime has been shown in crayfish, where the modulatory effect of serotonin on a neuronal circuit controlling escape behaviour depends on the individual's prior social experience, which affects the population of serotonin receptor subtypes expressed by the target neurons of serotonin in the escape circuitry [20]. Here, we have shown that socially induced global changes in behaviour differentially affect serotonin production across an anatomically and functionally heterogeneous population of regulatory neurons, with the neurons responsible for the serotonin signal that sets up the behavioural reconfiguration being distinct from those that manifest the long-term consequences of the altered behaviour. This strongly suggests that an understanding of experience-triggered behavioural states in other animals will likewise not be fully achieved by comparing expression profiles between normal subjects and subjects that manifest the chronic endpoint of behavioural change. Instead, our findings emphasize that ontogenetic time is of critical importance when seeking to understand the function of specific regulatory neurons in the reorganization of behaviour.

## Supplementary Material

ESM for Rogers & Ott

## Data accessibility

Raw measurements of cell body brightness and size and of total integrated neuropile brightness are available from the Dryad Digital Repository: http://dx.doi.org/10.5061/dryad.p5s65.
