# Supplementary material for: Differential activation of serotonergic neurons during short- and long-term gregarization of desert locusts
Source: Proc Biol Sci. 2015 Feb 7;282(1800):20142062. doi: 10.1098/rspb.2014.2062 (PMC4298206; doi:10.1098/rspb.2014.2062)
Supplement: ESM for Rogers & Ott [file rspb20142062supp1.pdf]

Supplemental Information

Supplemental Materials and Methods

Treatments to manipulate phase state

The intensity of serotonergic staining in the thoracic ganglia was compared across five different conditions: *(i) Long-term solitarious locusts* taken directly from the solitarious colony with no further treatment. *(ii) Long-term gregarious locusts* taken directly from the gregarious colony with no further treatment. *(iii) Solitarious locusts crowded together* with 20 gregarious locusts in a 15 cm × 19 cm × 13 cm plastic cage with a metal perch and without food for exactly 1 h in order to induce behavioural gregarisation. *(iv) Solitarious locusts given a mechanosensory touch stimulus directed to a hind femur* for exactly 1 h in order to induce behavioural gregarisation (1). Individual locusts were placed into clear plastic boxes (8 cm × 6 cm × 10 cm) with wire mesh at both narrow ends, through which a fine paintbrush was inserted to stroke the outside surface of the left hind femur for 5 s in each minute. *(v) Solitarious locusts given intense visual and olfactory stimuli from gregarious locusts* for 1 h but no physical contact in order to induce behavioural gregarisation (2). Individual locusts were placed in clear plastic pint glasses with double-layered mesh covering the top. The beakers were placed in a rearing cage containing 450–1,000 fifth-instar locust nymphs in the gregarious colony room for exactly 1 h.

Immunofluorescence staining

A zinc-formaldehyde fixative, modified from (3), was prepared by dissolving 4% paraformaldehyde at 80°C in 85 mM sodium acetate solution before adding 0.25% $ZnCl_2$ at room temperature (RT) and adjusting the pH to 6.5 with HCl. Immediately after behavioural observation in the arena, each locust was decapitated and the ventral thoracic body wall (including the thoracic nerve cord) was rapidly cut free and dropped into ice-cold fixative. All subsequent incubation and wash steps were carried out on an orbital shaker. After fixation for 24 h on ice, the preparations were washed for 3 × 1 h in 0.1 M Tris-HCl buffer (pH 7.4) on ice. The thoracic chain of ganglia was then dissected out under Tris buffer. To improve antibody penetration, the ganglion chains were treated with 20% dimethyl sulfoxide (DMSO) in methanol (4) for 1 h on ice, after which time they were brought to RT for another hour in the same solution, and transferred back into Tris buffer, which was changed once. This was followed by incubation in 1 mg·ml^{-1} collagenase (Sigma) + 1 mg·ml^{-1} hyaluronidase (Sigma) in 0.1 M phosphate-buffered saline (PBS; pH 7.4) at RT. The enzyme treatment was quenched after 35 minutes by a quick rinse and 2 × 15 minute washes in ice-cold 0.1 M phosphate buffer (PB; pH 7.4); re-fixation in 4% formaldehyde in PB for 60 minutes at RT to stabilise the tissue; and 4 × 30 minute washes in PB.

The ganglion chains were then incubated with 5% normal goat serum (NGS) in PBS containing 1% DMSO and 0.005% NaN_3 (PBS-D) for 2 h at RT, and then for 84 h at 4°C with a polyclonal rabbit anti-serotonin antiserum (Sigma, catalogue nr. S-5545) diluted 1:4,000 in PBS-D, 5% NGS. This was followed by 3 × 2 h washes in PBS-D at RT and incubation in Cy3-conjugated affinity-purified goat anti-rabbit IgG (H+L) antibodies (Jackson ImmunoResearch) diluted 1:200 in PBS-D, 5% NGS, for 60 h at 4°C. The chains were then passed through ascending grades of glycerol (to 70%) followed by 3 × 1 h in absolute ethanol before being transferred into methyl salicylate as described in (3), and finally mounted in DPX (BDH). Locusts from all treatment groups were included in each batch of immunohistochemical processing to ensure that all preparations were exposed to identical processing conditions.

Confocal microscopy

The immunofluorescence-stained ganglia were imaged in whole mount by confocal laser scanning microscopy using a 10× dry objective (numerical aperture 0.40). Stacks of confocal planes were captured at 1024 × 1024 pixel *xy*-resolution with a mechanical step size of 7 μm along the *z*-axis. All the metathoracic ganglia (n = 55) were imaged in one single session on a Leica SP1 microscope, and all the pro- and mesothoracic ganglia (n = 55 each) in a seperate single session on a Leica SP5 microscope. During a session, the imaging settings were kept strictly identical, and the ganglia were imaged in alternating order of treatment to rule out that a drift in microscope performance across the imaging session (several hours) might bias data from any one treatment group. Because the metathoracic ganglia were larger than the confocal image field of the SP1 microscope, two overlapping stacks were tiled to cover the anterior and posterior region of the ganglion and then merged (3). Furthermore, since this microscope had only 8 bit brightness resolution, each preparation was captured at two different photomultiplier gain settings (510 V and 685 V) and the resultant stacks merged to increase the dynamic range. The field of view and dynamic range (16 bit) of the SP5 confocal microscope were sufficient to capture an entire meso- or pro-thoracic ganglion in one stack. To avoid signal saturation, the photomultiplier gain was set such that even the brightest structures were well within the dynamic range of the detector. This was ensured by pre-screening many preparations using a look-up table (LUT) that highlights any saturated pixels, before committing to the gain that was then used throughout the acquisition session; and verified later by inspecting all scanned image stacks with the same LUT.

Characterization of the polyclonal 5HT antiserum

The immunogen used in generating the commercial 5HT antiserum that we used was 5HT conjugated to bovine serum albumin (BSA) through a condensation reaction with formaldehyde (FA) (5). The antiserum therefore contains antibody species against epitopes on BSA as well as against 5HT-derived epitopes. The latter antibodies typically have a ~1,000× higher affinity for the 5HT-FA derivative formed in the condensation reaction over free 5HT (6). To validate the specificity for 5HT in immunofluorescence staining in FA-fixed tissue, it was therefore necessary to preabsorb the antiserum with a BSA-FA-5HT conjugate, which eliminates all staining that represents 5HT. This would, however, also eliminate any staining from antibody species against epitopes on BSA that recognise BSA-like epitopes in locust. The effect of preabsorption with BSA-FA-5HT therefore also had to be compared with the effect of preabsorption with BSA alone.

The specificity of the 5HT antiserum was evaluated by two separate preabsorption tests. First we used an enzyme-linked immunosorbant assay (ELISA). Microplate wells were coated with BSA and then treated with FA in the absence or presence of 5HT, to mimic fixation of 5HT to tissue protein (6). Second, we performed whole-mount preparations of locust thoracic ganglia. We compared the binding of the full antiserum with the binding of the antiserum after preabsorbtion *(i)* with BSA alone; *(ii)* with additional BSA treated with FA in the absence of 5HT (BSA-FA); and *(iii)* with a mixture of BSA, BSA-FA and a 5HT-FA-BSA conjugate.

Conjugate preparation.—The BSA-FA-5HT conjugate was prepared following (5). BSA-FA was produced by replacing the 5HT creatinine sulphate solution in the conjugation reaction with water. Excess precipitated conjugate was removed by centrifugation (16,000 rcf for 1 h) and passing of the supernatants through a 0.45 μm pore-size syringe filter (Minisart, Sartorius). A Bradford assay against BSA standards gave concentrations of 1.05 mg·ml^{-1} and 11.6 mg·ml^{-1} for BSA-FA-5HT conjugate and BSA-FA, respectively.

Preabsorption of the 5HT antiserum.—The antiserum was diluted to 155 μg·ml^{-1} total protein (1:400) in 0.1 M PBS, pH 7.4, 0.005% NaN_3, either without any BSA *(i);* or with 10 mg·ml^{-1} BSA *(ii);* or with 10 mg·ml^{-1} BSA and 1 mg·ml^{-1} BSA-FA *(iii);* or additionally with up to

0.1 mg·ml^{-1} BSA-FA-5HT *(iv)* (final concentrations). After overnight agitation at 4°C, the mixtures were centrifuged at 16,000 rcf for 1 h at 2°C. The supernatants were diluted 1:10 to a final dilution of the antiserum of 1:4,000 in PBS containing either 0.05% Tween-20 (PBST) for ELISA; or 1% DMSO, 5% NGS and 0.005% NaN_3 for whole-mount immunofluorescence staining.

ELISA.—Nunc MaxiSorp 96-well plates (Thermo Fisher) were coated over night at 2°C with 125 µl of 10 mg·ml^{-1} BSA in 0.01 M PBS, pH 7.4, 0.005% NaN_3 and washed 3 × with PBS. To fix 5HT to the BSA coating, wells were loaded with 10 µl of 0.1 M 5HT creatinine sulphate in PBS (dissolved by sonication) and 115 µl of 3.7% FA in PBS (6). Control wells received 10 µl PBS and 115 µl 3.7% FA in PBS. The plates were incubated at RT for 1.5 h on a shaker. Wells that contained both 5HT and FA developed the faint yellow colour of the BSA-FA-5HT conjugate but there was no detectable colouration of the wells after washing (5 × in PBS, 3 × in PBST). One PBST wash was extended to at least 30 minutes to block non-specific binding of antibodies. Preabsorbed antisera (125 µl per well) were applied for 1.5 h at RT. After washing 4 × 5 minutes in PBST, wells were incubated for 1 h at RT with 125 µl affinity-purified alkaline phosphatase-conjugated goat anti-rabbit IgG (H+L) antibodies (Jackson ImmunoResearch) diluted 1:10,000 in TBST. The wells were washed (2 × PBST, 2 × PBS, 1 × water) and developed with 125 µl of 1 mg·ml^{-1} 4-nitrophenyl phosphate disodium salt hexahydrate in 0.1 M glycine-NaOH buffer, pH 10.4, 1 mM $ZnCl_2$, 1 mM $MgCl_2$ for 10–20 minutes after which the reactions were stopped by adding 125 µl 5% EDTA. The optical density at 405 nm was quantified on a Model 680 microplate reader (BioRad).

Supplemental Results

Characterisation by ELISA

We first established that preabsorbing the antiserum with increasing BSA-FA-5HT concentrations incrementally inhibits its binding to FA-fixed 5HT (Fig. S1). The highest optical density (OD) occurred with the whole antiserum on FA-5HT treated wells (n = 6 wells; Fig. S1*A*, magenta 'Δ' symbols) and was used as reference (relative OD, ROD = 1 ± 0.022 mean ± s.e.m.). As expected, preabsorption with BSA alone (Fig. S1*A*, green '+' symbols at 0 µg/ml BSA-FA-5HT) reduced binding substantially, reflecting the presence of antibodies against native BSA epitopes (ROD = 0.64 ± 0.013; n = 5 wells after removing one low outlier, t = 13.4, P = 2.92e-7). Co-preabsorption with increasing concentrations of 0.032–100 µg·ml^{-1} BSA-FA-5HT in the presence of a fixed concentration of 10 mg·ml^{-1} BSA led to a further reduction in OD in a sigmoid fashion, down to a very low residual ROD of 0.021 ± 0.00055 at 100 µg·ml^{-1} (Fig. S1*A*, green '+' symbols; n = 6), comparable to the ROD of controls without 5HT antiserum (Fig. S1*A*, blue '×' symbols). Between 0.32–100 µg·ml^{-1}, the reduction was approximately linear on a log-log scale (R^2 = 0.995, $F_{1,\,34}$ = 7055, P < 2.2e-16).

As expected, strong binding of the whole antiserum also occurred to BSA-coated wells treated with FA alone (Fig. S1*B*, magenta 'Δ' symbols; mean ROD = 0.74 ± 0.031, n = 4 wells); this was greatly reduced by preabsorbing the serum with BSA (Fig. S1*B*, green '+' symbols at 0 µg/ml BSA-FA-5HT; ROD = 0.033 ± 0.022, n = 4 wells; t = 18.5, P = 1.61e-6). Additional co-preabsorption with increasing BSA-FA-5HT concentrations caused a further log-log linear decrease of this very low residual binding (R^2 = 0.794, $F_{1,\,22}$ = 84.67, P = 5.36 × 10^{-9}). This suggested that the antiserum might contain some antibody species that bind preferentially to 5HT-unrelated BSA epitopes that have been modified by FA. We therefore included both BSA and FA-treated BSA (BSA-FA) in the positive control preabsorption for immunofluorescence staining.

Preabsorption of 5HT antiserum in immunofluorescence staining

We compared *(i)* staining obtained with the whole serum, *(ii)* staining after preabsorption of the antiserum with BSA and BSA-FA; and *(iii)* staining after additional co-preabsorption with BSA-FA-5HT conjugate (Fig. S2). The concentrations of the 5HT antiserum and of BSA and its derivatives were identical to those used in the second ELISA experiment. Conditions *(i)* and *(ii)* gave indistinguishable results: even those cells that were only weakly stained with the full antiserum (Fig. S2*A*) were unaffected by preabsorption with a combination of BSA and BSA-FA (Fig. S2*B*). By contrast, all staining was abolished after including BSA-FA-5HT conjugate in the preabsorption reaction (Fig. S2*C*).

Supplemental Discussion

Location and characterization of serotonergic neurones

The anatomy of the serotonergic system of locusts was first analysed using immunohistochemistry by Tyrer *et al.* (7) using material from both *Schistocerca gregaria* and the distantly related *Locusta migratoria*. Our data mostly agree with this previous study but we have detected more serotonergic somata in the thoracic ganglia. Tyrer *et al.* (7) noted the presence of smaller, more weakly stained neurones in the thoracic ganglia but did not describe them. The neurones in this earlier study were reconstructed from serial sections; improvements in immunostaining (3) and imaging techniques in the intervening years have allowed us to visualise neurones showing serotonin-like immunoreactivity more fully. The complete abolition of staining when the antibody was preabsorbed with BSA-FA-5HT, and the unaltered staining when preabsorbing it with BSA-FA strongly suggest that the target of the antibody in the CNS was indeed serotonin (Fig. S2).

Supplemental References

1. Simpson, S.J., Despland, E., Hagele, B., Dodgson, T. 2001 Gregarious behavior in desert locusts is evoked by touching their back legs. *Proc. Natl. Acad. Sci. USA* **98**, 3895–3897.

2. Roessingh, P., Bouaichi, A., Simpson, S.J. 1998 Effects of sensory stimuli on the behavioural phase state of the desert locust, Schistocerca gregaria. *J. Insect Physiol.* **44**, 883–893.

3. Ott, S.R. 2008 Confocal microscopy in large insect brains: zinc-formaldehyde fixation improves synapsin immunostaining and preservation of morphology in whole-mounts. *J. Neurosci. Methods* **172**, 220–230.

4. Dent, J.A., Polson, A.G., Klymkowsky, M.W. 1989 A whole-mount immunocytochemical analysis of the expression of the intermediate filament protein vimentin in *Xenopus. Development* **105**, 61–74.

5. Ranadive, N.S., Sehon, A.H. 1967 Antibodies to serotonin. *Can. J. Biochem.* **45**, 1701–1710.

6. Milstein, C., Wright, B., Cuello, A.C. 1983 The discrepancy between the cross-reactivity of a monoclonal antibody to serotonin and its immunohistochemical specificity. *Mol. Immunol.* **20**, 113–123.

7. Tyrer, N.M., Turner, J.D., Altman, J.S. 1984 Identifiable neurons in the locust central nervous system that react with antibodies to serotonin. *J. Comp. Neurol.* **227**, 313–330.

Supplemental Tables

Table S1. Results of independent contrasts of prothoracic serotonergic neurones, comparing mean immunofluorescence intensity with each treatment against long-term solitarious values. Significant contrasts are shown in bold.

	solitarious vs. gregarious			*vs. crowded*			*vs. hind leg touch*			*vs. sight+smell*		
Neurone	estimate	SE	*P*	estimate	SE	*P*	estimate	SE	*P*	estimate	SE	*P*
T1 cell 2	-2041	2022	0.318	88.14	2040	0.966	-1480	2042	0.472	-720.3	2372	0.763
T1 cell l3	-585.8	1241	0.639	592.8	1253	0.638	696.7	1253	0.581	415.3	1456	0.777
T1posterior group	228.3	526.1	0.666	369.2	530.8	0.490	332.7	531.2	0.534	790.2	617.0	0.206
T1posterolat. group	127.0	55.10	**0.026**	135.9	55.60	**0.018**	89.93	55.64	0.112	27.47	64.60	0.673
T1 cell 4[a]	1424	1026	0.172	1616	1035	0.125	475.8	1036	0.648	3206	1203	**0.010**
T1 cell 5	410.2	914.0	0.656	269.8	922.2	0.771	36.67	922.9	0.968	1914	1072	0.080
T1 lateral group[b]	179.7	138.4	0.200	462.2	139.6	**0.002**	120.6	139.7	0.392	362.2	162.3	**0.030**
T1 anterolat. group	-5393	3728	0.154	640.3	3762	0.866	-513.5	3765	0.892	12239	4373	**0.007**
T1 anterovent. group	-4498	2148	0.444	593.5	2560	0.818	-1465	2562	0.570	6479	2975	**0.034**
T1 anterior group	-2478	2476	0.322	573.1	2498	0.819	6776	2500	**0.009**	6520	2903	**0.029**

[a] One extreme outlier in the T1 cell 4 has been replaced with the mean value for the entire cell population.

[b] One extreme outlier in the T1 lateral group has been replaced with the mean value for the entire cell population.

Table S2. Results of independent contrasts of mesothoracic serotonergic neurones, comparing mean immunofluorescence intensity with each treatment against long-term solitarious values. Significant contrasts are shown in bold.

	solitarious vs. gregarious			*vs. crowded*			*vs. hind leg touch*			*vs. sight+smell*		
Neurone	estimate	SE	*P*	estimate	SE	*P*	estimate	SE	*P*	estimate	SE	*P*
T2 cell 1	-5749	1781	**0.002**	-1549	1752	0.381	-572.3	1758	0.746	1831	2065	0.379
T2 cell 2	-3196	1182	**0.009**	179.9	1163	0.878	-87.46	1166	0.941	652.1	1370	0.636
T2 posterior group	-13740	5912	**0.024**	3521	5822	0.548	4890	5841	0.407	9742	6861	0.162
T2 cell 3	139.3	135.2	0.308	418.8	133.0	**0.003**	308.1	133.4	**0.025**	457.9	156.7	**0.005**
T2 posterolat. group	719.9	2108	0.734	2810	2074	0.182	3201	2081	0.130	5197	2444	0.039
T2 cell 5	-83.10	82.09	0.316	18.03	80.77	0.824	1.300	81.02	0.987	167.8	95.17	0.084
T2 cell 4	-361.5	201.8	0.079	196.4	198.5	0.327	-31.56	199.1	0.875	1043	233.9	**<0.001**
T2 lateral pair	-132.8	82.21	0.113	30.84	80.88	0.705	100.0	81.14	0.224	325.6	95.30	**0.001**
T2 cell 6	-60.94	126.0	0.631	209.3	123.9	0.098	172.5	124.3	0.171	369.1	146.0	**0.015**
T2 anterovent. group[a]	-2718	2491	0.280	5316	2451	**0.035**	1680	2458	0.497	9461	2889	**0.002**
T2 anterior group	-8169	4026	**0.048**	8885	3961	**0.029**	3313	3974	0.408	13006	4668	**0.008**

[a] One extreme outlier for T2 anteroventral group has been replaced with the mean value for the entire cell population

Table S3. Results of independent contrasts of metathoracic serotonergic neurones, comparing mean immunofluorescence intensity with each treatment against long-term solitarious values. Significant contrasts are shown in bold.

	solitarious vs. gregarious			*vs. crowded*			*vs. hind leg touch*			*vs. sight+smell*		
Neurone	estimate	SE	*P*	estimate	SE	*P*	estimate	SE	*P*	estimate	SE	*P*
A3 cell 1	-218.2	68.46	**0.003**	-87.52	68.42	0.207	-123.8	68.93	0.079	87.69	80.08	0.279
A3 cell 2	-99.75	55.68	0.079	28.87	55.65	0.606	3.508	56.06	0.950	202.0	65.13	**0.003**
A3 cell 3	-177.0	41.09	**<0.001**	-16.11	41.07	0.697	-44.06	41.38	0.292	20.50	48.07	0.672
A2cell 1	-106.8	66.96	0.117	-60.05	66.92	0.374	-21.41	67.42	0.752	188.2	78.33	**0.020**
A2 cell 2	-65.92	45.1	0.150	-57.62	45.08	0.207	-39.67	45.41	0.387	200.5	52.76	**<0.001**
A2 cell 3	-94.64	24.11	**<0.001**	-4.682	24.10	0.847	-24.90	24.28	0.310	12.29	28.20	0.665
A1 cell 1	-219.3	51.43	**<0.001**	-76.88	51.40	0.141	-75.53	51.79	0.151	107.0	60.16	0.082
T3 lateral group	-931.4	266.6	**0.001**	113.0	266.4	0.673	153.2	268.4	0.571	465.4	311.8	0.142
T3 cell 1[a]	-113.2	49.26	**0.026**	4.884	49.23	0.921	0.764	49.60	0.988	73.29	57.62	0.209
T3 cell 2[b]	-0.526	3.022	0.863	-0.027	3.020	0.993	1.954	3.043	0.524	10.39	3.535	**0.005**
T3 cell 3	33.38	12.88	**0.013**	27.89	12.88	**0.035**	46.13	12.97	**0.001**	35.98	15.07	**0.021**
T3 cell 4	7.247	9.963	0.470	16.01	9.958	0.114	14.96	10.03	0.142	10.27	11.65	0.382
T3 anterior group	-107.4	61.05	0.085	65.01	61.02	0.292	73.74	61.47	0.236	62.94	71.41	0.382

[a] One extreme outlier for T3 cell 1 has been replaced with the mean value for the entire cell population.

[b] One extreme outlier for T3 cell 2 has been replaced with the mean value for the entire cell population.

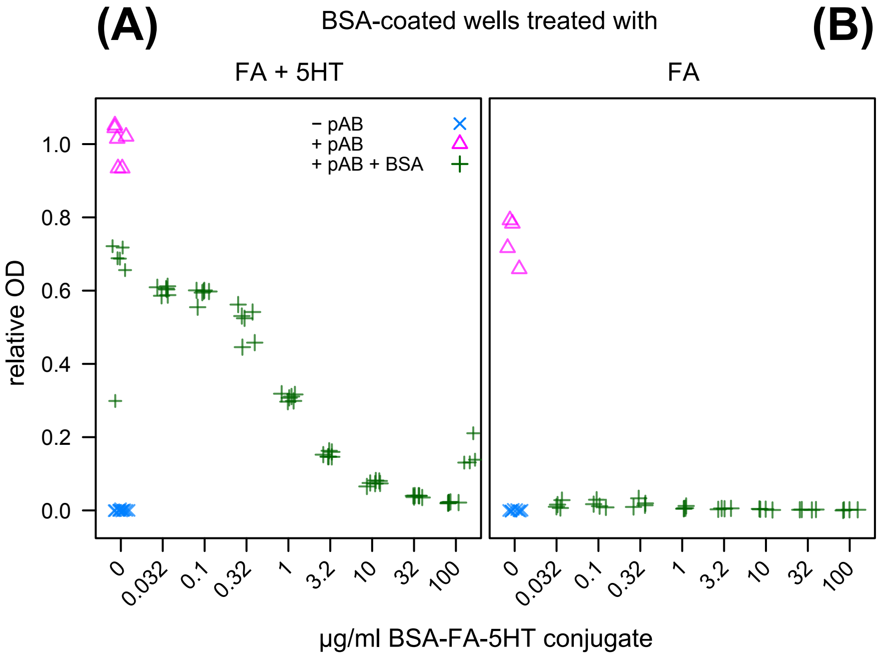

Figure S1. Characterisation of the 5HT antiserum by colorimetric ELISA. BSA-coated wells were treated with a mixture of 5HT and formaldehyde (FA; A) or with FA only (B). Each plot compares the relative optical density (OD) seen with the full 5HT antiserum (magenta 'Δ') with that seen after co-preabsorbing the antiserum with increasing concentrations of BSA-FA-5HT conjugate (x-axis) in the presence of 10 $mg{\cdot}ml^{-1}$ BSA (green '+'); blue '×' is binding in the absence of the 5HT antiserum. OD is expressed relative to the mean for whole antiserum in wells treated with both 5HT and FA.

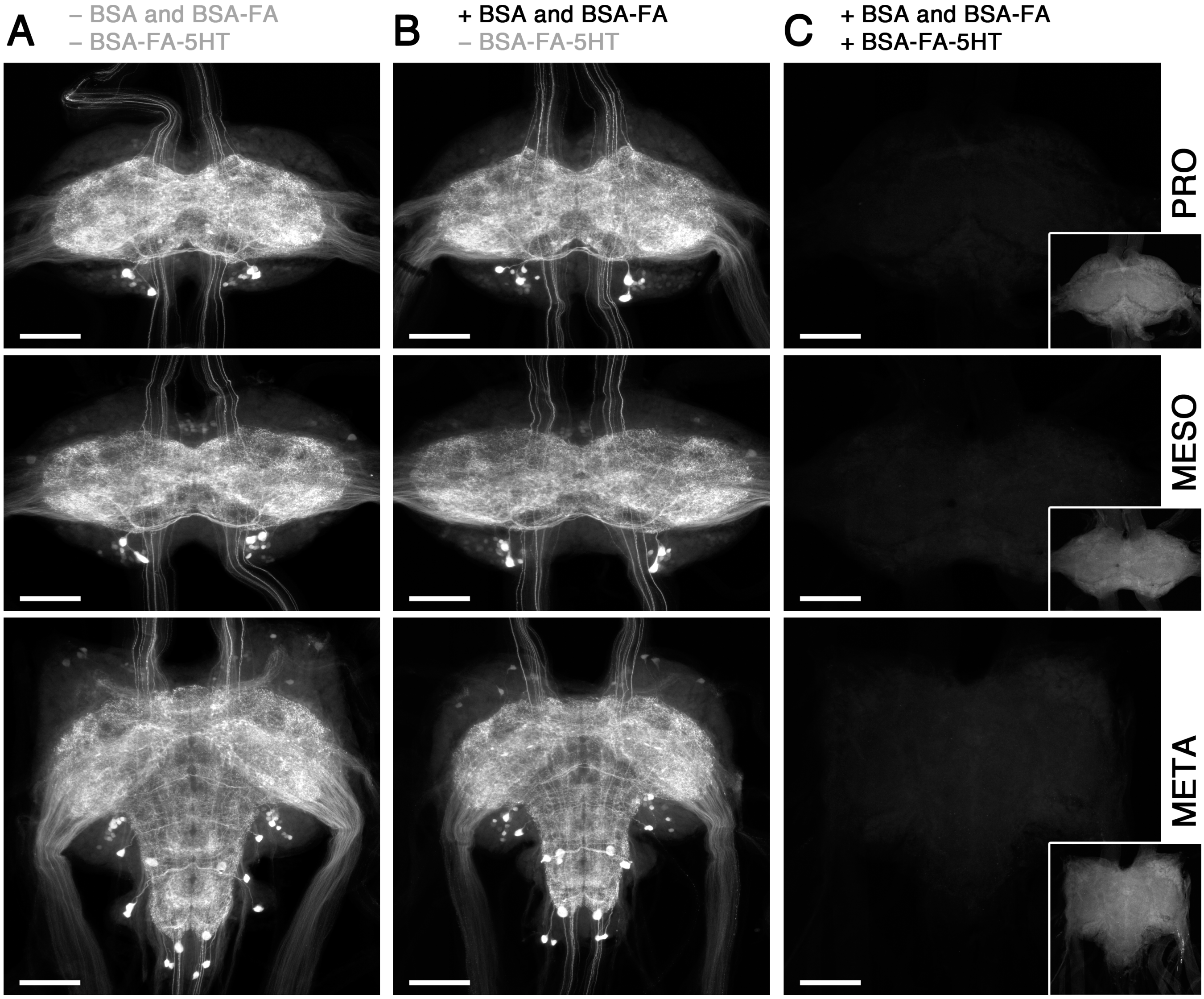

Figure S2. Characterisation of anti-5HT immunofluorescence (IF) staining in locust thoracic ganglia by preabsorption of the 5HT antiserum. Confocal image stacks were maximum-projected in the *z*-axis and log-transformed to increase the simultaneous visibility of faintly and intensely stained structures. PRO, prothoracic ganglion; MESO, mesothoracic ganglion; META, metathoracic ganglion. (A) No preabsorption of the 5HT antiserum. (B) Preabsorption of the antiserum (155 $\mu g \cdot ml^{-1}$ total protein) with 10 $mg \cdot ml^{-1}$ BSA and 1.2 $mg \cdot ml^{-1}$ formaldehyde-treated BSA (BSA-FA) has no effect on the intensity or pattern of IF. (C) Additional co-preabsorption with 100 $\mu g \cdot ml^{-1}$ BSA-FA-5HT conjugate abolishes all IF. In the insets, the brightness gain has been digitally increased to make the ganglia visible.

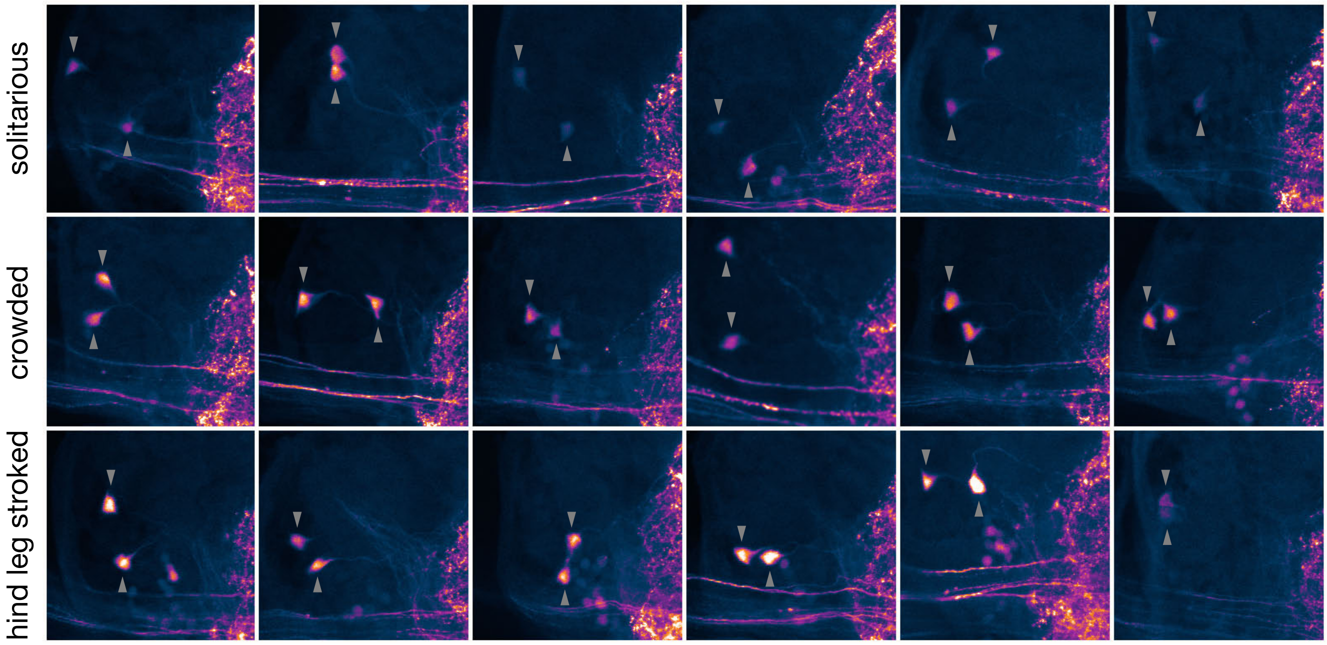

Figure S3. The somata (grey arrowheads) of the pair of neurones (T3-3 and T3-4) in the metathoracic ganglion that show increased serotonin expression following exposure to gregarising stimuli. Each image is the arithmetic sum of eleven consecutive confocal optical sections encompassing the two somata, divided by the total integrated brightness of the metathoracic neuropile in the respective preparation. All images are shown on the same pseudo-colour intensity scale. Each row shows the first six in the set of twelve preparations used in the statistical analysis in this paper. Top row: uncrowded long-term solitarious locusts; middle row: long-term solitarious locusts that had been behaviourally gregarised by crowding for 1 hour; bottom row: uncrowded long-term solitarious locusts that had been behaviourally gregarised by stroking their hind leg for 1 hour.
